# KOTMIN13, a Korean herbal medicine alleviates allergic inflammation in vivo and in vitro

**DOI:** 10.1186/s12906-016-1155-4

**Published:** 2016-06-06

**Authors:** Eujin Lee, Sun-Gun Kim, Na-Young Park, Hyo-Hyun Park, Kyu-Tae Jeong, Jongkeun Choi, In-Hae Lee, Hwadong Lee, Keuk-Jun Kim, Eunkyung Lee

**Affiliations:** Research and Development Division, National Development Institute of Korean Medicine, Gyeongsan, 712-260 Republic of Korea; Department of Cosmetic Science, Chungwoon University, Chungnam, 350-701 Republic of Korea; Department of Clinical Pathology, Daekyeung University, Gyeongsan, 712-719 Republic of Korea

**Keywords:** Airway hyperresponsiveness (AHR), Bronchoalveolar lavage fluids (BALF), Bone-marrow derived mast cells (BMMC), Passive cutaneous anaphylaxis (PCA), Degranulation, Leukotriene C_4_ (LTC_4_)

## Abstract

**Background:**

The ethanol extract of KOTMIN13, composed of *Inula japonica* Flowers, *Trichosanthes kirilowii* Semen, *Peucedanum praeruptorum* Radix, and *Allium macrostemon* Bulbs, was investigated for its anti-asthmatic and anti-allergic activities.

**Methods:**

The anti-asthmatic effects of KOTMIN13 were evaluated on ovalbumin (OVA)-induced murine asthma model. Anti-allergic properties of KOTMIN13 in bone-marrow derived mast cells (BMMC) and passive cutaneous anaphylaxis (PCA) in vivo were also examined.

**Results:**

In asthma model, KOTMIN13 effectively suppressed airway hyperresponsiveness induced by aerosolized methacholine when compared to the levels of OVA-induced mice. KOTMIN13 treatment reduced the total leukocytes, eosinophil percentage, and Th2 cytokines in the bronchoalveolar lavage fluids in OVA-induced mice. The increased levels of eotaxin and Th2 cytokines in the lung as well as serum IgE were decreased by KOTMIN13. The histological analysis shows that the increased inflammatory cell infiltration and mucus secretion were also reduced. In addition, the degranulation and leukotriene C_4_ production were inhibited in BMMC with IC_50_ values of 3.9 μg/ml and 1.7 μg/ml, respectively. Furthermore, KOTMIN13 treatment attenuated mast-mediated PCA reaction.

**Conclusions:**

These results demonstrate that KOTMIN13 has anti-asthmatic and anti-allergic effects in vivo and in vitro models.

## Background

The allergic reaction is biphasic. The immediate reaction occurs within minutes caused by release of preformed mediators from basophils and mast cells upon allergen exposure. The late phase allergic reaction is caused by mobilization and accumulations of inflammatory cells, resulting further release of pharmacologically active mediators, sustaining the allergic response and promoting the late allergic response [1]. Asthma is a chronic inflammatory disorder of the airways associated with airway hyperresponsiveness (AHR) and airflow obstruction. Activation of Th2 cells in the airway is responsible for the pathogenesis of this disease. Th2 cells orchestrate the recruitment and activation of mast cells and eosinophils through the release of Th2 cytokines such as IL-4, IL-5 and IL-13, which are the primary effector cells of the allergic response.

Mast cells are widely distributed throughout human respiratory tract and found in alveoli walls [2]. They are major effector cells that play a role in allergic inflammation. When activated through IgE-dependent or IgE-independent ways, mast cells release preformed mediators (histamine, proteases, and proteglycans) from their granules, lipid mediators (leukotrienes (LTs) and prostaglandins (PGs)) derived from arachidonic acid, and synthesize cytokines and chemokines [2, 3].

The medicinal plants have been used in traditional medicine to treat allergic diseases and their activities have been demonstrated [4–6]. A Korean herbal medicine named KOTMIN13, composed of *Inula japonica* Thunberg, *Trichosanthes kirilowii Maximowicz* var. *japonica kitamura*, *Peucedanum praeruptorum* Dunn, and *Allium macrostemon* Bge, has been used for the purpose of anti-allergic and anti-asthmatic treatment in an oriental clinic, but its activities have not been investigated. In the present study, we investigated the effects of KOTMIN13 on the treatment of asthma in vivo model as well as allergic response by measuring inflammatory mediators in bone-marrow derived mast cells (BMMC) and passive cutaneous anaphylaxis (PCA) in mice.

The results demonstrated that KOTMIN13 attenuated ovalbumin (OVA)-induced airway inflammation by reducing AHR, leukocyte infiltration, the levels of Th2 cytokine, eotaxin, and serum IgE production, as well as mucus secretion in a murine asthma model. Furthermore, we showed that these effects were associated in part with the suppression of activated mast cells by inhibiting degranulation and eicosanoid production in BMMC as well as PCA in vivo.

## Methods

### Plant materials

Herbs (*Inula japonica* Flowers, *Trichosanthes kirilowii* Semen, *Peucedanum praeruptorum* Radix, and *Allium macrostemon* Bulbs) were purchased from Humanherb (Gyeongsan, Korea) and authenticated by Dr. H. Lee, an herbalist. A voucher specimen has been deposited at the National Development Institute of Korean Medicine. The herbs were mixed according to the ratio of combination (10:8:8:5), extracted with 30 % ethanol at a ratio of 1:10 (*w/v*) and then refluxed for 24 h at 60 °C. The extracted solution was filtered and the solvent evaporated under vacuum at 40 °C (Eyela, Tokyo, Japan), before being freeze-dried to obtain a concentrated extract (15.4 % yield).

### Murine asthmatic model and treatment

Six weeks old female BALB/c mice (16–20 g) were obtained from Koateck (Seoul, Korea) and fed with laboratory chow (Purina, Seoul, Korea) and water ad libitum. Animals were acclimatized in a specific pathogen-free animal facility under the conditions of 20–22 °C, 40–60 % relative humidity, and 12 h/12 h (light/dark) cycle at least for 7 days. Mice were sensitized by intraperitoneal administration on days 0 and 14 with 20 μg/ml of OVA in PBS mixed with equal volumes of alum (1 mg) as an adjuvant. The mice were challenged from day 22 to 24 with 1 % OVA in PBS or PBS using a nebulizer (NE-U17, OMRON, Tokyo, Japan). Mice were randomly divided into 7 groups groups (*n* = 6 per group): NC (negative control group, PBS sensitization and challenge), OVA (OVA sensitization and challenge positive group), KOTMIN13 (50, 100, 200 mg/kg), montelukast (Mont, 20 mg/kg), and dexamethasone (Dex, 1 mg/kg). KOTMIN13, Mont, or Dex was treated per orally 10 times at every 12 h from 1 day before the first challenge to the last challenge. Mice care and experimental procedures were conducted with the approval of the animal care committee of National Development Institute of Korean Medicine (Approval No. KOTMIN-2015-001 for asthma).

### AHR measurement

AHR to aerosolized methacholine (Sigma, St. Louis, MO, USA) was measured in the plethysmograph chamber (Emka Technologies, Paris, France) according to the manufacture’s protocol. In brief, mice were stabilized in the chamber for 10 min and then exposed to aerosolized saline (1 min) as a control. Mice were then challenged every 20 min with aerosolized methacholine. Increasing doses of aerosolized methacholine were administered and enhanced pause (Penh) was measured over the subsequent 5 min as an index of airway obstruction.

### Analysis of total cells and eosinophils in bronchoalveloar lavage fluid (BALF)

BALF was obtained as described previously [7] and immediately centrifuged (2 min, 4 °C, 160 g). After removing the supernatant, the cells were resuspended in 0.5 ml of PBS. After total cell counting, the suspended cells were spun onto glass microscope slides (Shandon Cytospin 4, Thermo Scientific, Kalamazoo, MI, USA) and stained with hematoxylin and eosin (H&E). The number of eosinophils was determined by counting at least 100 cells in each of four different locations, and data were expressed as a percentage of total leukocytes.

### Enzyme-liked immunosorbent assays (ELISA)

The levels of eotaxin and cytokines in BALF and lung homogenate supernatant were quantified using ELISA according to the manufacturer’s instructions (R&D Systems, Inc., Minneapolis, MN, USA). Blood was collected from mice via cardiac puncture and serum was obtained by centrifugation (1000 g for 10 min at 4 °C) and stored at −70 °C. Total serum IgE was measured by using Mouse IgE ELISA kit (BD Biosciences, San Diego, CA, USA).

### Histological analysis of lung tissue

The lungs were removed and fixed with 10 % (*v/v*) formaldehyde prior to embedding in paraffin. The sections of fixed paraffin tissues were cut (4 μm thick), deparaffinized and stained with H&E, and then periodic acid Schiff reagent (PAS) to measure leukocyte accumulation and mucus secretion, respectively.

### BMMC preparation

Bone marrow cells from male BALB/cJ mice were cultured in RPMI 1640 media (2 mM L-glutamine, 100 U/ml penicillin, 100 μg/ml streptomycin) and 10 % fetal calf serum from (Hyclone, South Logan, UT, USA) containing 20 % pokeweed mitogen-stimulated spleen condition medium (Sigma). After 3 weeks, BMMC were used for assays.

### Determination of β-hexosaminidase (hex) release, LTC_4_ generation and local anaphylaxis

The release of β-hex was quantified by spectrophotometirc method as described previously [8]. For LTC_4_ determination, BMMC at a cell density of 1 × 10^6^ cells/ml were sensitized overnight with anti-dinitrophenyl (DNP) IgE (500 ng/ml) and seeded in 96 well plate. After pre-incubated with KOTMIN13 for 30 min, BMMC were stimulated with DNP-human serum albumin (HSA, 100 ng/ml) for 15 min and all reactions were stopped by centrifugation at 120 g at 4 °C for 5 min, and then the supernatants were immediately used for LTC_4_ determination. The level of LTC_4_ was determined using EIA kit (Cayman Chemical, Ann Arber, MI, USA) accordance with the manufacturer’s protocols.

### PCA test

The experimental method for PCA was described previously [8]. In short, mouse anti-DNP IgE (80 ng, Sigma) were intradermally injected into ears of 7-week old ICR male mice, followed 24 h later by oral administration of KOTMIN13 (50, 100, and 200 mg/kg) or fexofenadine-HCl (fexo), a histanime H1 receptor antagonist (Korea Pharma, Seoul, 50 mg/kg). The mice were intravenously challenged with DNP-HSA (60 μg, Sigma) in PBS containing 1 % (*w/v*) Evans blue and ears were removed to determine the amount of dye extravasation (at 630 nm). Mice care and experimental procedures were performed under the approval by Animal Care Committee of National Development Institute of Korean Medicine (Approval No. KOTMIN-2015-006 for PCA).

### HPLC analysis

The chromatographic system was composed of a shimadzu LC-2AP Binary HPLC pump and a SPD-20A Photodiode Array Detector (Shimadzu, Corp., Kyoto, Japan). Detection and quantification were performed using Empower software. The separation was carried out on a Waters Sunfire C_18_ column (250 mm × 4.6 mm, 5 μm) at a column temperature of 40 °C. The injection volume was 20 μL for a sample. The detection wavelength was 330 nm. The mobile phase consisted of Solvent A (0.3 % aqueous acetic acid (*v/v*)) and Solvent B (acetonitrile) with gradient elution at the flow rate of 1.0 ml/min: 20 % Solvent B at 0 min, 40 % B at 40 min, 20 % B at 50 min, 20 % B at 60 min.

### Statistical analysis

The data are expressed as mean ± SEM. Statistical significance was determined by one-way ANOVA followed by Duncan’s multiple range tests. A value of *p* < 0.05 was considered statistically significant.

## Results

### Effects of KOTMIN13 on AHR

AHR to methacholine (doses of 2.5, 5, 20, 30, 40 mg/kg) was examined 18 h after the final OVA challenge. The responsiveness of the control mice to methacholine was weak as shown in Table 1. However, methacholine administration significantly increased the Penh values in OVA-induced and OVA-challenged mice compared with the controls. KOTMIN13 treatment significantly reduced the Penh values in a dose dependent manner. Treatment of mice with 200 mg/kg of KOTMIN13 strongly diminished the increased AHR and resulted in similar dose–response curves of AHR to that of the Dex.

**Table 1:** The effect of KOTMIN13 on AHR

Group	Concentration	Methacholine-induced Penh level				
		2.5 mg/kg	5 mg/kg	20 mg/kg	30 mg/kg	40 mg/kg
NC		1.56 ± 0.13	1.73 ± 0.03	2.15 ± 0.10	3.28 ± 0.25	3.65 ± 0.30
OVA		4.31 ± 0.26^##^	4.71 ± 0.20^##^	6.47 ± 0.36^##^	10.50 ± 1.00^###^	12.00 ± 0.57^###^
KOTMIN13	50 mg/kg	3.53 ± 0.13^*^	3.87 ± 0.25^*^	4.99 ± 0.42^*^	6.97 ± 0.37^**^	7.36 ± 0.88^**^
	100 mg/kg	2.99 ± 0.23^**^	3.38 ± 0.18^**^	5.01 ± 0.28^*^	5.96 ± 0.39^***^	6.98 ± 0.26^***^
	200 mg/kg	2.34 ± 0.06^***^	2.69 ± 0.23^***^	3.70 ± 0.19^***^	5.16 ± 0.18^***^	5.17 ± 0.48^***^
Montelukast	20 mg/kg	3.03 ± 0.20^**^	3.43 ± 0.18^**^	5.34 ± 1.34^*^	5.92 ± 0.28^***^	6.49 ± 0.23^***^
Dexamethasone	1 mg/kg	2.00 ± 0.05^***^	2.30 ± 0.20^***^	4.09 ± 0.12^**^	5.16 ± 0.29^***^	5.13 ± 0.37^***^

Data were expressed as the mean ± SEM. ^##^ *P* <0.01 and ^###^ *P* <0.001 compared with the negative control (NC); *P <0.05, **P <0.01, and ***P <0.001 compared with the OVA group

### Effects of KOTMIN13 on total cells and eosinophils in BALF

**Fig. 1:**
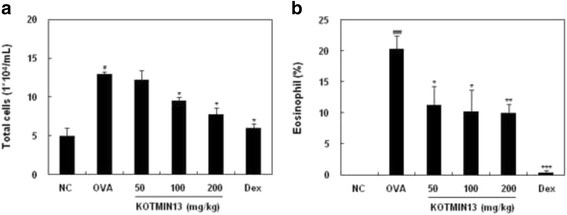
Effects of KOTMIN13 on the recruitment of leukocytes in BALF. BALF was obtained after aerosolized OVA inhalation challenge. **a** Total leukocytes and eosinophils from the BALF were counted. **b** The eosinophils were expressed as a percentage of the total leukocytes. Values represent mean ± SEM. ^#^ *P* < 0.05 and ^###^ *P* < 0.001 compared with the negative control (NC); *P < 0.05, **P < 0.01, and ***P < 0.001 compared with the OVA (OVA sensitized/challenged) group. Dex, dexamethasone

To evaluate the anti-asthmatic effect of KOTMIN13 in an in vivo model, the total number of leukocytes and the percentage of eosinophils in the BALF were determined. The total leukocytes in the BALF of the OVA-induced lung tissues were increased as compared to control mice. However, KOTMIN13 (100 and 200 mg/kg) inhibited the OVA-induced recruitment of total leukocytes into the airway (Fig. 1a). The number of eosinophils in the BALF of mice was determined as a percentage of the total number of cells and KOTMIN13 treatment decreased the eosinophil percentage recruited into the airways of the OVA-induced mice (Fig. 1b).

### Effects of KOTMIN13 on eotaxin, cytokines, and serum IgE

**Fig. 2:**
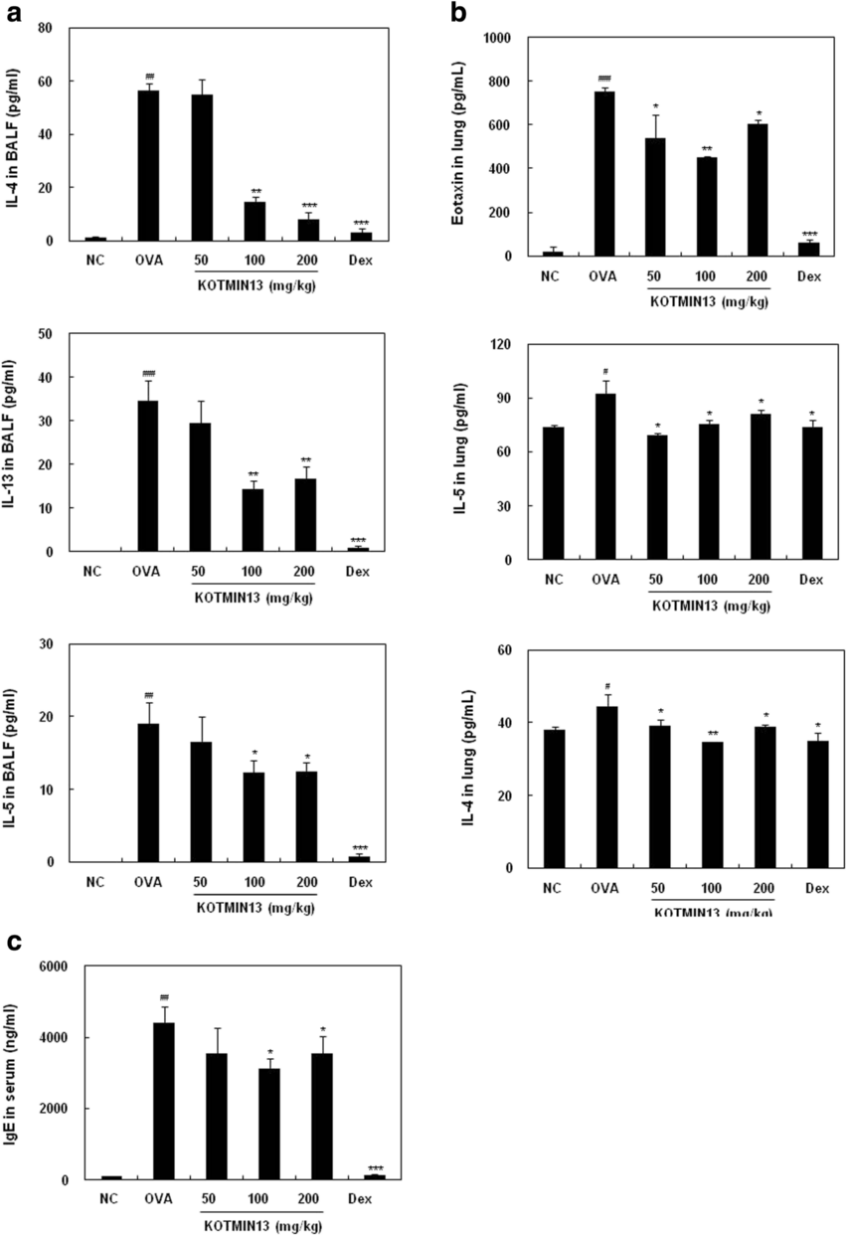
The effect of KOTMIN13 on the levels of eotaxin, cytokines, and serum IgE. The levels of Th2 cytokines (IL-4, IL-5, and IL-13) and eotaxin in (**a**) BALF and (**b**) lung, and (**c**) serum IgE were determined using ELISA. Values represent mean ± SEM. ^#^ *P* < 0.05, ^##^ *P* < 0.01, and ^###^ *P* < 0.001 compared with the negative control (NC); *P < 0.05, **P < 0.01, and ***P < 0.001 compared with the OVA (OVA sensitized/challenged) group. Dex, dexamethasone

The levels of Th2 cytokines and eotaxin were measured in the BALF and lung homogenate supernatant using ELISA. As shown in Fig. 2a, OVA sensitization and challenge induced significant elevation of IL-4, IL-5, and IL-13 in the BALF. KOTMIN13 treatment (at doses of 100 and 200 mg/kg) decreased the concentration of these cytokines. The levels of eotaxin and cytokines including eotaxin, IL-4, and IL-5 in the lung of mice were also determined (Fig. 2b). Eotaxin level was higher in OVA-induced mice than that in control mice. KOTMIN13 treatment at dose of 100 and 200 mg/kg effectively inhibited the increased eotaxin in the lung of OVA-induced mice. The levels of IL-4 and IL-5 were increased in the OVA-induced asthmatic mice when compared to control mice and KOTMIN13 treatment decreased the levels of IL-4 and IL-5 in the lung. Therefore, KOTMIN13 treatment attenuated the eotaxin and Th2 cytokine levels in both BALF and the lung of OVA-induced mice. We also determined whether KOTMIN13 modulated the levels of serum IgE in OVA-induced mice. As shown in Fig. 2c, the levels of serum IgE were significantly increased in OVA-induced mice when compared to control mice. However, KOTMIN13 treatment at 100 and 200 mg/kg led to a decrease in the serum levels of IgE.

### Histological analysis of lung tissue

**Fig. 3:**
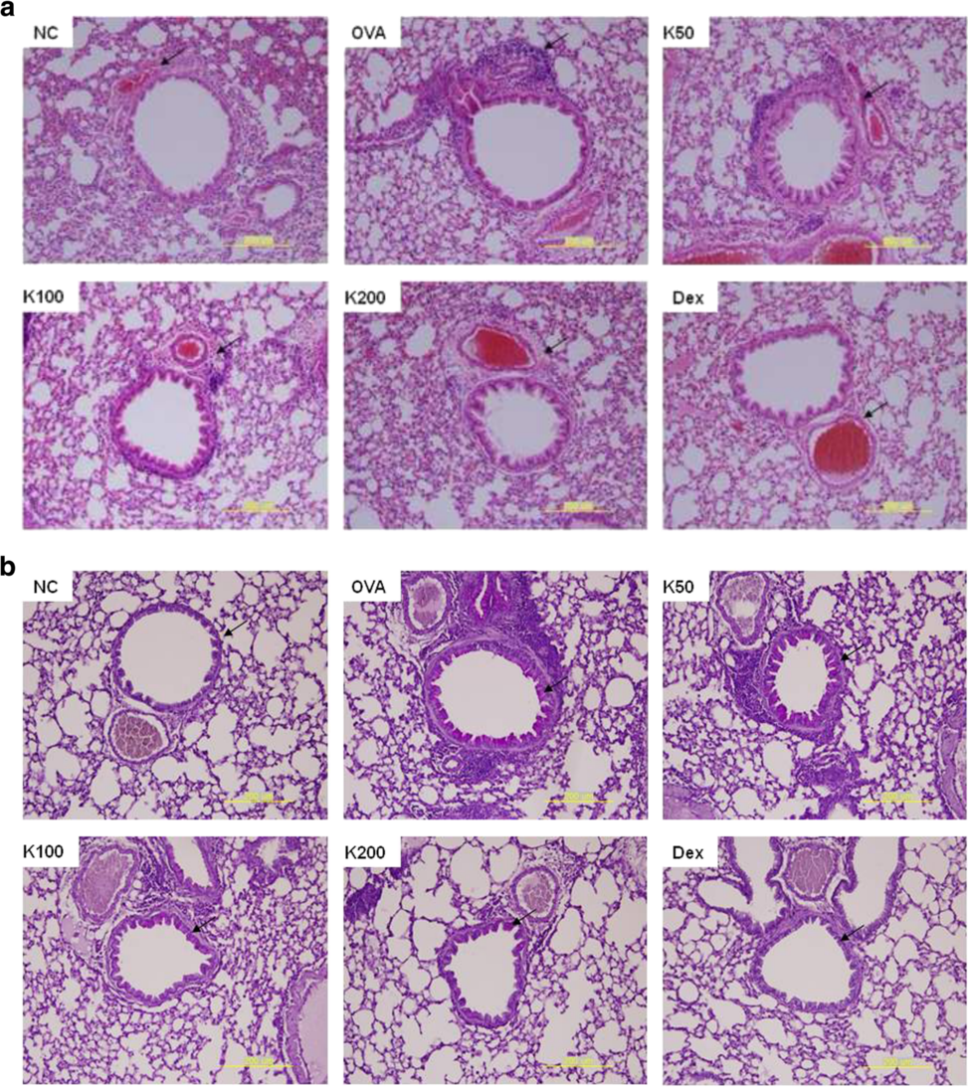
Histological analysis of lung tissue. Histological analysis of lung tissue was conducted. Lung sections of control mice and asthmatic mice treated with KOTMIN13 or Dex were stained with (**a**) H&E or (**b**) PAS. Arrows indicates inflammatory cells and mucus production. Values represent mean ± SEM. NC, negative control; OVA, OVA sensitized/challenged; K, KOTMIN13; Dex, dexamethasone. Magnification 200×

To examine the inhibitory effects of KOTMIN13 on the histological changes in the OVA-induced asthma model, lung tissues were stained with H&E and PAS staining solution. The infiltration of inflammatory cells into perivascular and peribronchial areas was observed in OVA-challenged mice. KOTMIIN13 treatment reduced the degree of inflammatory cell infiltration in the perivascular and peribronchial areas (Fig. 3a). Mucus secretion within the bronchi of the lungs was also examined. An increase in intensity of PAS staining was observed in the lung tissues of OVA-induced mice and KOTMIN13 treatment reduced mucus production in lung tissues when compared with control (Fig. 3b). As expected, Dex significantly decreased perbronchial and perivascular lung inflammation.

### Effect of KOTMIN13 on β-hex release, LTC_4_ generation, and local anaphylaxis

**Fig. 4:**
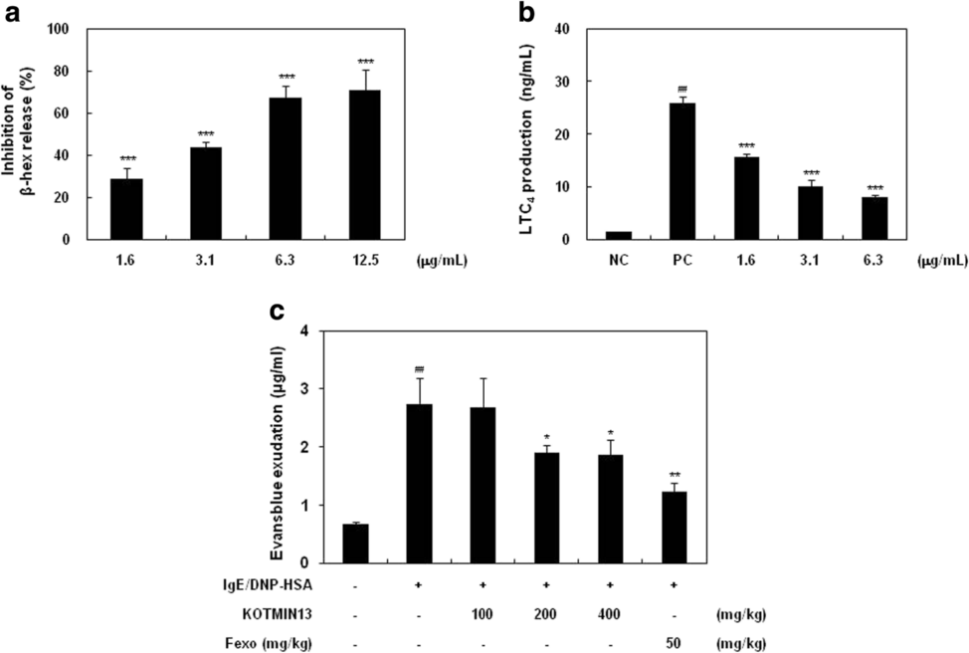
Effect of KOTMIN13 on β-hex release, LTC_4_ production, and PCA. BMMC were sensitized with anti-DNP IgE, treated with KOTMIN13, and then challenged with DNP-HSA. **a** β-hex and **b** LTC_4_ released into the supernatant were determined. **c** In PCA test, mice were sensitized by intradermal injection of IgE (80 ng) into mouse ears and intravenously challenged with DNP-HSA (60 μg) containing Evans blue after oral administration of KOTMIN13 for 1 h. Ears were removed for measuring the amount of dye extravasation. Values represent mean ± SEM. ^##^ *P* < 0.01 compared with the negative control (NC, anti-DNP IgE); *P < 0.05, **P < 0.01, and ***P < 0.001 compared with the positive control (PC, anti-DNP IgE/DNP-HSA)

Since histamine release by activated mast cells parallels the release of β-hex, the effect of KOTMIN13 on degranulation was determined by β-hex release. As shown in Fig. 4a, KOTMIN13 treatment reduced the release of β-hex (IC_50_ = 3.9 μg/ml) in activated BMMC. The inhibitory activity of KOTMIN13 on the LTC_4_ production was shown in a dose-dependent manner with an IC_50_ value of 1.7 μg/ml (Fig. 4b). These results suggested that KOTMIN13 has inhibitory activities on the release of preformed and synthesized ecosanoid mediators in activated mast cells. We also examined the anti-allergic activity of KOTMIN13 on PCA. An oral administration of KOTMIN13 1 h before injection of antigen inhibited the mast cell-mediated PCA reaction (31 % at 200 mg/kg and 32 % at 400 mg/kg), indicating that KOTMIN13 exerts anti-allergic effects through the inhibition of mast cell degranulation.

### HPLC analysis

**Fig. 5:**
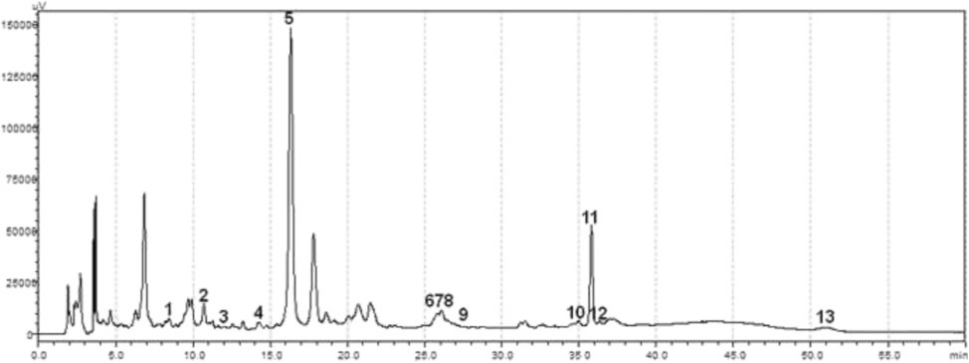
Representative HPLC-UV chromatograms of KOTMIN13 extract and its components. 1, (−)Secoisolariciresinol; 2, Isoquercitrin; 3, Cucurbitacin; 4, Anthricin; 5, 1,5-*di*-*O*-caffeoylquinic acid; 6, Quercitrin; 7, Quercetin; 8, Luteolin; 9, Praeruptorin A; 10, 1-*O*-acetylbritannilactone; 11, Britanin; 12, (−)Pinoresinol; 13, Tomentosin

More than 20 compounds from KOTMIN13 were purified and identified. Among them, the identification of thirteen compounds was based on the retention times and the UV spectrum in comparison with authentic standards at a wavelength of 330 nm. The 13 compound profile of KOTMIN13 analyzed via HPLC (Fig. 5) showed that one phenyl propanoid (secoisolariciresinol), four flavonoids (isoquercitrin, quercitrin, quercetin, and luteolin), four terpenes (cucurbitacin, 1-*O*-acetylbritannilactone, britanin, and tomentosin), two lignans (anthricin and pinoresinol), one polyphenol (1,5-*di*-*O*-caffeoylquinic acid), and one coumarin (praeruptorin A) were isolated and purified from KOTMIN13. The chemical structures were determined by comparison of their NMR spectral data with standards.

## Discussion

Asthma is a chronic inflammatory disorder of airway in which many cells and cellular components play a role, in particular, mast cells, eosinophils, T cells, neutrophils, and epithelial cells [9]. Although corticosteroids with potent anti-inflammatory and anti-allergy activities remain the major therapy for allergic diseases, long-term medication can lead to serious side effects and complications [10]. Various plant extracts have been used in traditional medicine to treat allergic diseases and their activities have been demonstrated [4–6]. Therefore, many researchers in Asia have tried to generate data to support herbal medicine for usage as natural anti-inflammatory products because it is necessary to demonstrate that herbal medicine is safe and effective.

KOTMIN13 is composed of four herbs: *Inula japonica* Flowers, *Trichosanthes kirilowii* Semen, *Peucedanum praeruptorum* Radix, and *Allium macrostemon* Bulbs. It is modified from Guaruhaebaebaekju-tang, which has frequently used for asthma treatment in traditional herbal medicine. Although KOTMIN13 has been used for treatment of anti-inflammatory and anti-allergic diseases such as asthma and allergic rhinitis in a local clinic, there are still no valid data of its anti-allergic effect and the mechanism underlying the anti-asthmatic effect of KOTMIN13. In previous study, we demonstrated that KOTMIN13 inhibits the production of inflammatory mediators including NO, PGE_2_, and pro-inflammatory cytokines that are mediated through NF-kB and MAPK activity inhibition in LPS-induced RAW 264.7 cells. In addition, KOTMIN13 ameliorated the development of phorbol ester 12-*O*-tetradecanoylphorbol-13-acetate-induced ear edema and carrageenan-induced paw edemas in acute inflammatory edema models (unpublished data).

Since various mediators released during allergic inflammation play a critical role in AHR development [11], AHR is a hallmark of clinical symptom for asthma. Increased Th2 cytokines such as IL-4, IL-5, and IL-13 play a vital role in asthmatic responses. IL-4 increases IgE production and participates in the initiation of Th2 inflammatory responses. IL-5 plays a crucial role in AHR by mobilizing and activating eosinophils [12]. IL-13 has been shown to induce mucus hypersecretion and AHR in murine asthma models in which cysteinyl-LTs might be causative agents of AHR [13]. In the present study, our results demonstrated that KOTMIN13 treatment alleviated AHR, decreased total leukocyte recruitment, and the production of Th2 cytokines in the BALF and lungs of OVA-induced mice, which was consistent with the results of the histological analysis. Therefore, the reduced Th2 cytokine production in this experiment led to low levels of serum IgE as well as decreased leukocyte infiltration and mucus secretion into the lung tissue of asthmatic mice.

Histamine is one of the most potent mediators in the acute phase of immediate hypersensitivity and triggers acute symptoms due to its rapid effects on the bronchial and smooth muscle cells [14]. Cysteinyl LTs also affect inflammatory cell recruitment, mucus production, airways remodeling, and pulmonary vascular leakage [15]. In this study, the effect of KOTMIN13 on β-hex release from stimulated BMMC was determined and revealed that KOTMIN13 inhibited β-hex release in a dose dependent manner. In addition, LTC_4_ production from stimulated BMMC was also reduced by KOTMIN13 treatment. These results indicate that KOTMIN13 suppresses the degranulation and LTC_4_ production which attenuate inflammatory response with asthma and other inflammatory disease. PCA is one of the most important in vivo models of immediate hypersensitivity in local allergic reaction. In this study, PCA reaction was induced by the injection of IgE and antigen and oral administration of KOTMIN13 decreased the PCA reaction in a dose dependent manner, indicating its role in the prevention or treatment of mast cell-mediated allergic reactions such as asthma.

Among thirteen compounds isolated from KOTMIN13, sesquiterpene lactons such as 1-*O*-acetylbritannilactone, britanin, and tomentosin from Inulae flos have been proven to exhibit anti-inflammatory activities in others and our previous studies [16–19]. Flavonols (quercetin, quercitrin, and isoquercetin) from Inulae flos function as anti-inflammatory and anti-allergic activities [20–25]. Luteolin also attenuates AHR in OVA-induced mice and inhibited mast cell-mediated allergic inflammation [26, 27]. Other compounds from Inulae flos such as 1,5-*di*-*O*-dicaffeoylquinic acid were identified in HPLC analysis. Recently, several other anti-inflammatory compounds from Inulae flos were also isolated [28, 29]. Praeurptorin A isolated from *Peucedanum praeruptorum* Radix and *Allium macrostemon* Bulbs has showed anti-inflammatory effects in LPS-stimulated RAW 264.7 cells, as well as suppressed OVA-induced airway inflammation and remodeling in mice [30–32]. Two compounds, cucurbitacin and pinoresinol from *Trichosanthes kirilowii* semen were identified by HPLC analysis. Cucurbitacin B has anti-inflammatory activities in vitro and in vivo and pinoresinol attenuates inflammatory responses of microglia on the production of inflammatory mediators by the inhibition of ERK and NF-kB activities [33–35]. Although compounds such as praeurptorin B, praeurptorin C, 3,4-dihydroxy benzoic acid, di-*O*-caffeoylquinic acid, and miquelianin were not in the HPLC chromatogram, they were isolated from KOTMIN13. The active compounds mentioned above are involved in the anti-allergic responses of KOTMIN13, and other compounds derived from KOTMIN13 may have anti-allergic activity as well.

## Conclusion

In summary, our study suggests that KOTMIN13 is an alternative natural drug in the OVA-induced airway inflammation by decreasing AHR, leukocyte infiltration, Th2 cytokines, and IgE production, as well as mucus secretion in a mouse model of asthma. In addition, anti-allergic activities of KOTMIN13 could be in part mediated by decreasing pharmacologically active mediators from mast cells.

## Abbreviations

β-hex, β-hexosaminidase; AHR, airway hyperresponsiveness; BALF, bronchoalveolar lavage fluid; BMMC, bone-marrow derived mast cells; Dex, dexamethasone; DNP, dinitrophenyl; ELISA, enzyme-liked immunosorbent assays; ERK, extracellular signal regulated kinase; Fexo, fexofenadine; H&E, hematoxylin and eosin; HSA, human serum albumin; LT, leukotriene; MAPK, mitogen-activated protein kinase; Mont, montelukast; NF-kB, nuclear factor-kappa B; NO, nitric oxide; OVA, ovalbumin; PAS, periodic acid Schiff; PCA, passive cutaneous anaphylaxis; Penh, enhanced pause; PG, prostaglandin
